# Bis[μ-3,5-bis­(pyridin-2-yl)pyrazolato]bis­[(hexa­fluoro­phosphato)copper(II)]

**DOI:** 10.1107/S1600536813018813

**Published:** 2013-07-13

**Authors:** Akio Mishima, Nagisa Katsuta, Midori Furusyou, Akira Fuyuhiro, Satoshi Kawata

**Affiliations:** a Department of Chemistry, Faculty of Science, Fukuoka University, Nanakuma, Jonan-ku, Fukuoka 814-0180, Japan; b Department of Chemistry, Graduate School of Science, Osaka University, Toyonaka, Osaka 560-0043, Japan

## Abstract

The title dinuclear complex mol­ecule, [Cu_2_(C_13_H_9_N_4_)_2_(PF_6_)_2_], lies about an inversion center. The Cu^II^ atom shows a square-pyramidal coordination geometry with the basal plane formed by four N atoms of the two bis-chelating 3,5-bis­(pyridin-2-yl)pyrazolate ions and with one F atom of the hexa­fluoro­phosphate ion in the apical position. Mol­ecules are stacked in a column along the *a* axis through C—H⋯F hydrogen bonds. The columns are further linked by other C—H⋯F hydrogen bonds, forming a three-dimensional network.

## Related literature

For metal complexes of 3,5-bis­(2-pyrid­yl)pyrazole, see: Klingele *et al.* (2009 ▶); Yoneda, Adachi, Hayami *et al.* (2006 ▶); Yoneda, Adachi, Nishio *et al.* (2006 ▶); Ishikawa *et al.* (2010 ▶); Mishima *et al.* (2011 ▶); Washizaki *et al.* (2012 ▶). For an example of a coordinated hexa­fluoro­phosphate ion, see: Noro *et al.* (2011 ▶).
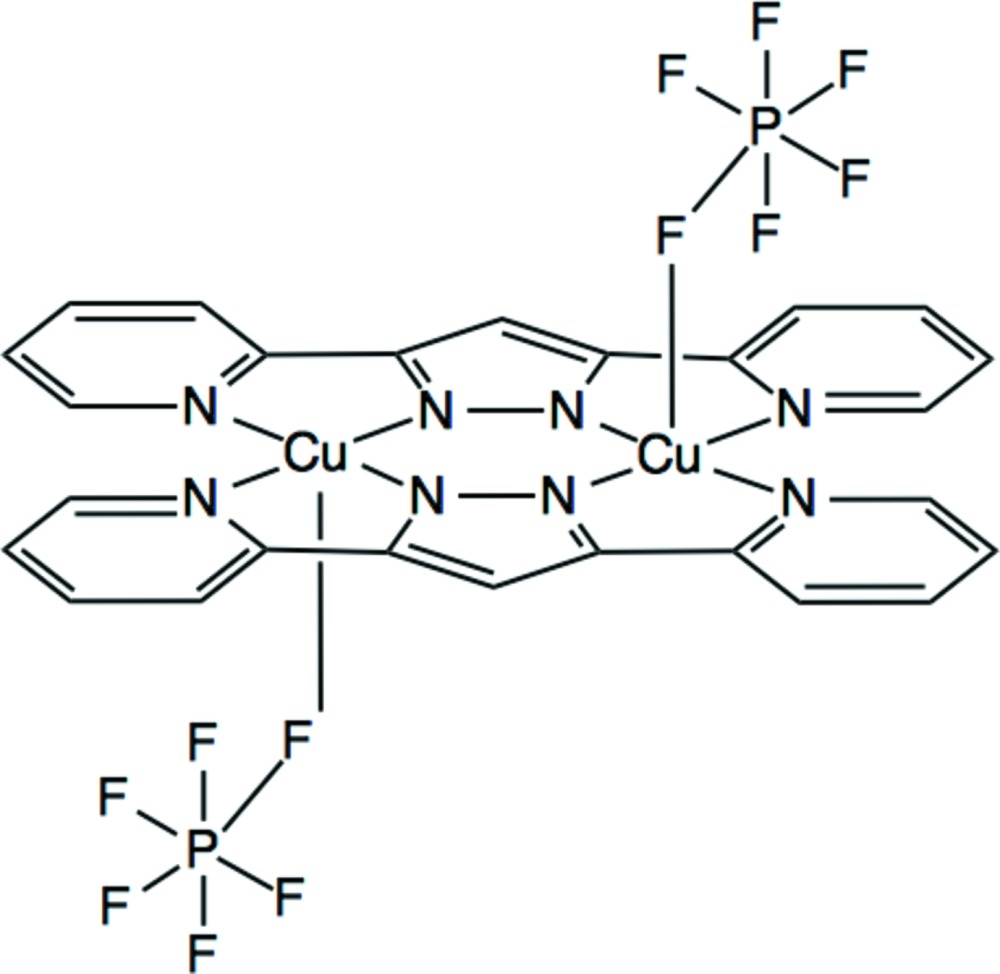


## Experimental

### 

#### Crystal data

[Cu_2_(C_13_H_9_N_4_)_2_(F_6_P)_2_]
*M* *_r_* = 859.52
Monoclinic, 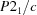

*a* = 6.3558 (4) Å
*b* = 21.2388 (14) Å
*c* = 10.9252 (9) Å
β = 95.753 (2)°
*V* = 1467.36 (18) Å^3^
*Z* = 2
Mo *K*α radiation
μ = 1.67 mm^−1^
*T* = 200 K
0.50 × 0.15 × 0.10 mm

#### Data collection

Rigaku R-AXIS RAPID diffractometer
Absorption correction: multi-scan (*ABSCOR*; Higashi, 1995 ▶) *T*_min_ = 0.603, *T*_max_ = 0.845
23498 measured reflections
3364 independent reflections
3035 reflections with *F* ^2^ > 2σ(*F* ^2^)
*R*_int_ = 0.028

#### Refinement

*R*[*F* ^2^ > 2σ(*F* ^2^)] = 0.027
*wR*(*F* ^2^) = 0.074
*S* = 1.05
3364 reflections
226 parameters
H-atom parameters constrained
Δρ_max_ = 0.44 e Å^−3^
Δρ_min_ = −0.19 e Å^−3^

### 

Data collection: *RAPID-AUTO* (Rigaku, 2002 ▶); cell refinement: *RAPID-AUTO*; data reduction: *RAPID-AUTO*; program(s) used to solve structure: *Il Milione* (Burla *et al.*, 2007 ▶); program(s) used to refine structure: *SHELXL97* (Sheldrick, 2008 ▶); molecular graphics: *CrystalStructure* (Rigaku, 2010 ▶); software used to prepare material for publication: *CrystalStructure*.

## Supplementary Material

Crystal structure: contains datablock(s) I, global. DOI: 10.1107/S1600536813018813/is5289sup1.cif

Structure factors: contains datablock(s) I. DOI: 10.1107/S1600536813018813/is5289Isup2.hkl

Supplementary material file. DOI: 10.1107/S1600536813018813/is5289Isup3.cdx

Additional supplementary materials:  crystallographic information; 3D view; checkCIF report

## Figures and Tables

**Table 1:** Selected bond lengths (Å)

Cu1—F1	2.4027 (14)
Cu1—N1^i^	2.0698 (15)
Cu1—N2^i^	1.9393 (16)
Cu1—N3	1.9405 (15)
Cu1—N4	2.0577 (17)

Symmetry code: (i) 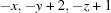.

**Table 2:** Hydrogen-bond geometry (Å, °)

*D*—H⋯*A*	*D*—H	H⋯*A*	*D*⋯*A*	*D*—H⋯*A*
C3—H3⋯F3^ii^	0.95	2.31	3.257 (3)	175
C11—H7⋯F2^iii^	0.95	2.54	3.451 (3)	162
C12—H8⋯F5^iv^	0.95	2.60	3.456 (3)	150
C13—H9⋯F3^iv^	0.95	2.52	3.226 (3)	131

Symmetry codes: (ii) 
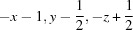
; (iii) 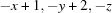; (iv) 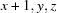.

**Fig. 1.**
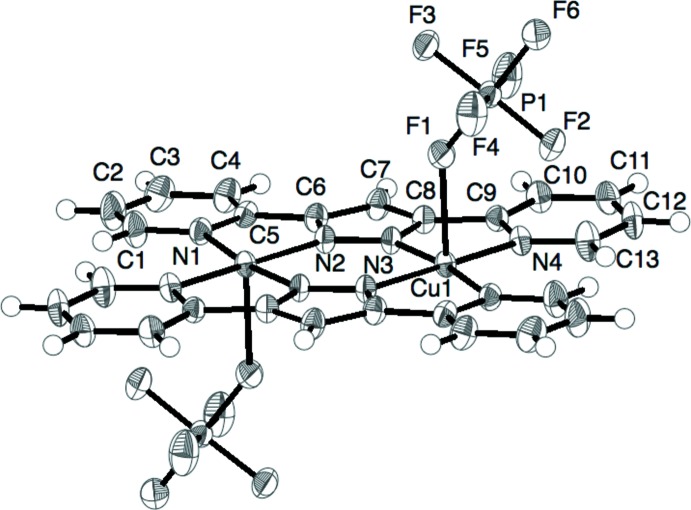
An ORTEP drawing of the title complex, showing 50% probability displacement ellipsoids.

**Fig. 2.**
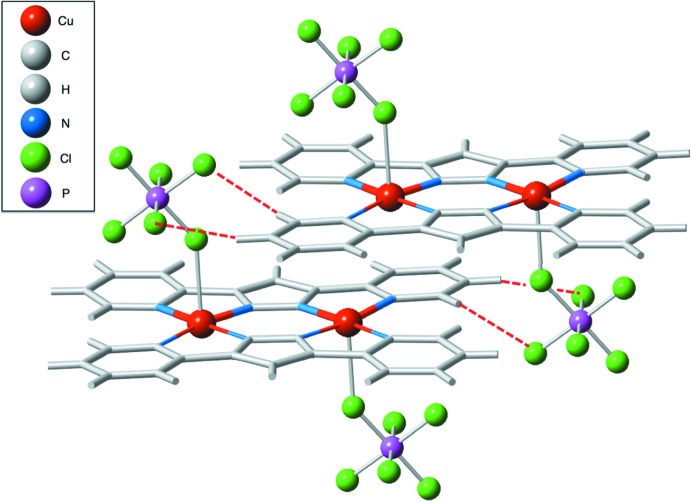
A fragment of one-dimensional structure of the title compound. Hydrogen bonds are shown as dashed lines.

**Fig. 3.**
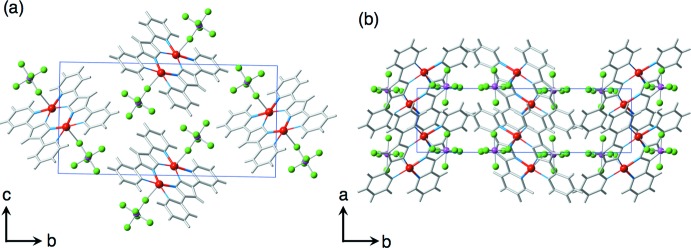
Packing structures of the title complex viewed along the a axis (a) and the c axis (b).

## supplementary crystallographic information

### Comment

3,5-Bis(2-pyridyl)pyrazole [Hbpypz] can be used to construct of a series of mononuclear, dinuclear and polynuclear complexes as it is well-known due to the versatile properties of the ligand (Klingele *et al.*, 2009). The Hbpypz has four N donors by deprotonation; the two N atoms in a pyrazole moiety and two N atoms in pyridine moieties. This ligand can bind to metal ions by behaving as a bidentate or as a tetradentate ligand and would be possible to form various coordination modes (Yoneda, Adachi, Hayami *et al.*, 2006; Yoneda, Adachi, Nishio *et al.*, 2006). In particular, two bpypz^-^ ions form a planar dinuclear complex by chelating two metal ions at equatorial position (Washizaki *et al.*, 2012). The dinuclear complex has unique coordination sites at the apical positions, which can trap ions hardly to coordinate. We have previously reported the dinuclear complex with coordinated hydrogensulfate ions at the apical sites (Mishima *et al.*, 2011). The complex consists of a planer dinuclear complex and two hydrogensulfate ions, and forms a 1D chain with methanol molecules by hydrogen-bonding interactions.

The title planer dinuclear Cu^II^ complex with two PF_6_^-^ ions has a similar structure to the above complex. The basal plane in the complex is formed by four N donors of two deprotnated tetradentate bridging bpypz^-^ ligands. Cu—N distances are Cu—N1 2.0698 (15) Å, Cu—N2 1.9393 (16) Å, Cu—N3 1.9405 (15) Å, and Cu—N4 2.0577 (17) Å. Cu^II^ ions are each penta-coordinated by occupying PF_6_^-^ ion at apical positions in the opposite direction and form a near ideal square-pyramidal coordination environment with t value of 0.068. The distance of Cu—F1 is 2.4027 (14) Å. To the best of our knowledge, the crystal structure report of PF_6_^-^ coordinated Cu^II^ complex is only a few examples (Noro *et al.*, 2011). The adjacent dinuclear complexes are stacked in columns through a weak π–π stacking interaction between pyridyl and pyrazol rings of the bpypz^-^ ions (centroid-centroid distance 3.879 Å) and C—H···F hydrogen bonds between the bpypz^-^ and the PF_6_^-^ ions (Table 2). The C—H···F interactions are expected to be weak because of the low acidity of C—H system. However, the interatomic distances are in close contact; the distances of C—H···F bond are H1···F3 2.689 Å, H2···F4 2.666 Å, H8···F5 2.603 Å and H9···F3 2.522 Å. The supramolecular structure results from C—H···F bonds between adjacent columns. The distances between the columns are H3···F3 2.308 Å, H7···F2 2.536 Å and H5···F8 2.634 Å.

### Experimental

A methanolic solution of Cu(AcO)_2_.H_2_O (5ml, 20 mmol dm^-3^ ) was transferred to a glass tube, and then a methanolic solution of Hbpypz (5ml, 20 mmol dm^-3^), NaPF_6_ (5 ml, 10 mmol dm^-3^) were poured into the glass tube without mixing the solutions. Purple crystals began to format ambient temperature within one week. Yield: 14 mg (54 %). Elemental analysis (%) calcd for C_26_H_18_N_8_F_12_P_2_Cu_2_ : C 36.33, H 2.11, N 13.04; found: C 36.29, H 2.13, N 13.04.

### Refinement

The C-bound hydrogen atoms in the bpypz^-^ ion were placed at calculated positions (C—H = 0.95 Å) and were treated as riding on their parent atoms with *U*_iso_(H) = 1.2*U*_eq_(C).

### Crystal data

**Table:**

[Cu_2_(C_13_H_9_N_4_)_2_(F_6_P)_2_]	*F*(000) = 852.00
*M**_r_* = 859.52	*D*_x_ = 1.945 Mg m^−^^3^
Monoclinic, *P*2_1_/*c*	Mo *K*α radiation, λ = 0.71075 Å
Hall symbol: -P 2ybc	Cell parameters from 17353 reflections
*a* = 6.3558 (4) Å	θ = 3.2–27.5°
*b* = 21.2388 (14) Å	µ = 1.67 mm^−^^1^
*c* = 10.9252 (9) Å	*T* = 200 K
β = 95.753 (2)°	Block, purple
*V* = 1467.36 (18) Å^3^	0.50 × 0.15 × 0.10 mm
*Z* = 2	

### Data collection

**Table:**

Rigaku R-AXIS RAPID diffractometer	3035 reflections with *F*^2^ > 2σ(*F*^2^)
Detector resolution: 10.000 pixels mm^-1^	*R*_int_ = 0.028
&yen;w scans	θ_max_ = 27.5°
Absorption correction: multi-scan (*ABSCOR*; Higashi, 1995)	*h* = −7→8
*T*_min_ = 0.603, *T*_max_ = 0.845	*k* = −27→27
23498 measured reflections	*l* = −14→14
3364 independent reflections	

### Refinement

**Table:**

Refinement on *F*^2^	Secondary atom site location: difference Fourier map
*R*[*F*^2^ > 2σ(*F*^2^)] = 0.027	Hydrogen site location: inferred from neighbouring sites
*wR*(*F*^2^) = 0.074	H-atom parameters constrained
*S* = 1.05	*w* = 1/[σ^2^(*F*_o_^2^) + (0.0407*P*)^2^ + 0.8933*P*] where *P* = (*F*_o_^2^ + 2*F*_c_^2^)/3
3364 reflections	(Δ/σ)_max_ = 0.005
226 parameters	Δρ_max_ = 0.44 e Å^−^^3^
0 restraints	Δρ_min_ = −0.19 e Å^−^^3^
Primary atom site location: structure-invariant direct methods	

### Special details

**Table:**

Refinement. Refinement was performed using all reflections. The weighted R-factor (wR) and goodness of fit (S) are based on F^2^. R-factor (gt) are based on F. The threshold expression of F^2^ > 2.0 sigma(F^2^) is used only for calculating R-factor (gt).

### Fractional atomic coordinates and isotropic or equivalent isotropic displacement parameters (Å^2^)

**Table:**

	*x*	*y*	*z*	*U*_iso_*/*U*_eq_	
Cu1	0.24888 (3)	1.033789 (9)	0.417882 (19)	0.02189 (8)	
P1	0.03196 (7)	1.13684 (2)	0.14887 (5)	0.02699 (12)	
F1	0.0266 (2)	1.09594 (7)	0.27373 (13)	0.0462 (4)	
F2	0.28337 (18)	1.14060 (7)	0.17046 (13)	0.0442 (4)	
F3	−0.22096 (18)	1.13202 (6)	0.13015 (12)	0.0396 (3)	
F4	0.0145 (3)	1.19979 (7)	0.22615 (15)	0.0542 (4)	
F5	0.0487 (3)	1.07334 (7)	0.07324 (15)	0.0563 (4)	
F6	0.0329 (3)	1.17709 (8)	0.02601 (13)	0.0538 (4)	
N1	−0.4133 (3)	0.88408 (7)	0.53595 (15)	0.0270 (4)	
N2	−0.0897 (3)	0.93626 (7)	0.45127 (14)	0.0253 (3)	
N3	0.0774 (3)	0.95817 (7)	0.39846 (15)	0.0252 (3)	
N4	0.4018 (3)	0.98801 (7)	0.28636 (15)	0.0267 (4)	
C1	−0.5796 (4)	0.85849 (11)	0.5849 (2)	0.0407 (5)	
C2	−0.6626 (4)	0.80010 (12)	0.5503 (3)	0.0457 (6)	
C3	−0.5719 (4)	0.76610 (11)	0.4628 (3)	0.0422 (5)	
C4	−0.4009 (4)	0.79120 (10)	0.4109 (2)	0.0361 (5)	
C5	−0.3257 (3)	0.85002 (9)	0.44940 (18)	0.0267 (4)	
C6	−0.1453 (3)	0.88030 (9)	0.40028 (18)	0.0270 (4)	
C7	−0.0116 (3)	0.86473 (9)	0.31120 (19)	0.0308 (4)	
C8	0.1266 (3)	0.91584 (9)	0.31413 (17)	0.0267 (4)	
C9	0.3078 (3)	0.93294 (9)	0.24875 (17)	0.0259 (4)	
C10	0.3792 (4)	0.89636 (10)	0.15631 (19)	0.0345 (5)	
C11	0.5527 (4)	0.91622 (11)	0.1003 (2)	0.0379 (5)	
C12	0.6503 (4)	0.97141 (10)	0.1374 (3)	0.0394 (5)	
C13	0.5705 (4)	1.00590 (10)	0.2299 (3)	0.0395 (5)	
H1	−0.6429	0.8815	0.6461	0.0489*	
H2	−0.7809	0.7839	0.5868	0.0549*	
H3	−0.6258	0.7258	0.4381	0.0506*	
H4	−0.3360	0.7685	0.3498	0.0433*	
H5	−0.0140	0.8282	0.2608	0.0370*	
H6	0.3096	0.8581	0.1319	0.0413*	
H7	0.6039	0.8919	0.0366	0.0454*	
H8	0.7705	0.9858	0.1005	0.0473*	
H9	0.6386	1.0443	0.2547	0.0474*	

### Atomic displacement parameters (Å^2^)

**Table:**

	*U*^11^	*U*^22^	*U*^33^	*U*^12^	*U*^13^	*U*^23^
Cu1	0.02108 (12)	0.02079 (12)	0.02492 (13)	−0.00163 (8)	0.00777 (8)	0.00105 (8)
P1	0.0235 (3)	0.0286 (3)	0.0298 (3)	0.00159 (18)	0.00704 (18)	0.00549 (18)
F1	0.0339 (7)	0.0609 (9)	0.0441 (8)	0.0039 (6)	0.0048 (6)	0.0282 (7)
F2	0.0238 (6)	0.0595 (9)	0.0501 (8)	−0.0005 (6)	0.0075 (5)	0.0071 (7)
F3	0.0243 (6)	0.0452 (7)	0.0494 (8)	0.0015 (5)	0.0050 (5)	0.0164 (6)
F4	0.0554 (9)	0.0404 (8)	0.0704 (10)	−0.0047 (7)	0.0235 (8)	−0.0159 (7)
F5	0.0550 (9)	0.0471 (8)	0.0710 (11)	−0.0066 (7)	0.0268 (8)	−0.0213 (8)
F6	0.0451 (8)	0.0709 (10)	0.0458 (8)	−0.0083 (7)	0.0071 (6)	0.0299 (7)
N1	0.0240 (7)	0.0287 (8)	0.0289 (8)	−0.0056 (6)	0.0053 (6)	0.0000 (7)
N2	0.0256 (7)	0.0233 (8)	0.0282 (8)	−0.0040 (6)	0.0094 (6)	−0.0015 (6)
N3	0.0246 (7)	0.0246 (8)	0.0279 (8)	−0.0027 (6)	0.0099 (6)	−0.0008 (6)
N4	0.0275 (8)	0.0237 (8)	0.0307 (8)	0.0021 (6)	0.0118 (7)	0.0025 (6)
C1	0.0360 (11)	0.0469 (13)	0.0420 (12)	−0.0155 (10)	0.0168 (9)	−0.0086 (10)
C2	0.0388 (12)	0.0486 (13)	0.0521 (14)	−0.0233 (11)	0.0162 (10)	−0.0052 (11)
C3	0.0375 (11)	0.0326 (11)	0.0567 (14)	−0.0141 (9)	0.0057 (10)	−0.0042 (10)
C4	0.0332 (10)	0.0275 (10)	0.0486 (13)	−0.0046 (8)	0.0086 (9)	−0.0067 (9)
C5	0.0220 (8)	0.0260 (9)	0.0324 (10)	−0.0017 (7)	0.0036 (7)	0.0014 (8)
C6	0.0256 (9)	0.0232 (9)	0.0330 (10)	−0.0025 (7)	0.0069 (7)	−0.0016 (7)
C7	0.0279 (9)	0.0277 (10)	0.0381 (11)	−0.0038 (8)	0.0095 (8)	−0.0078 (8)
C8	0.0250 (8)	0.0271 (9)	0.0288 (9)	−0.0005 (7)	0.0073 (7)	−0.0040 (7)
C9	0.0229 (8)	0.0291 (9)	0.0265 (9)	0.0016 (7)	0.0061 (7)	0.0015 (7)
C10	0.0322 (10)	0.0392 (11)	0.0333 (10)	−0.0023 (9)	0.0100 (8)	−0.0092 (9)
C11	0.0368 (11)	0.0457 (12)	0.0335 (11)	0.0041 (10)	0.0155 (9)	−0.0042 (9)
C12	0.0369 (11)	0.0407 (12)	0.0449 (13)	−0.0004 (9)	0.0247 (10)	0.0031 (9)
C13	0.0407 (12)	0.0303 (10)	0.0517 (13)	−0.0069 (9)	0.0257 (10)	−0.0022 (9)

### Geometric parameters (Å, º)

**Table:**

Cu1—F1	2.4027 (14)	C3—C4	1.382 (4)
Cu1—N1^i^	2.0698 (15)	C4—C5	1.388 (3)
Cu1—N2^i^	1.9393 (16)	C5—C6	1.463 (3)
Cu1—N3	1.9405 (15)	C6—C7	1.394 (3)
Cu1—N4	2.0577 (17)	C7—C8	1.395 (3)
P1—F1	1.6205 (16)	C8—C9	1.460 (3)
P1—F2	1.5937 (13)	C9—C10	1.386 (3)
P1—F3	1.6033 (13)	C10—C11	1.380 (4)
P1—F4	1.5911 (17)	C11—C12	1.368 (4)
P1—F5	1.5908 (16)	C12—C13	1.384 (4)
P1—F6	1.5919 (16)	C1—H1	0.950
N1—C1	1.346 (3)	C2—H2	0.950
N1—C5	1.354 (3)	C3—H3	0.950
N2—N3	1.342 (3)	C4—H4	0.950
N2—C6	1.345 (3)	C7—H5	0.950
N3—C8	1.346 (3)	C10—H6	0.950
N4—C9	1.358 (3)	C11—H7	0.950
N4—C13	1.345 (3)	C12—H8	0.950
C1—C2	1.385 (4)	C13—H9	0.950
C2—C3	1.371 (4)		
			
F1—Cu1—N1^i^	86.91 (6)	C1—C2—C3	119.1 (3)
F1—Cu1—N2^i^	89.26 (6)	C2—C3—C4	119.1 (3)
F1—Cu1—N3	95.51 (6)	C3—C4—C5	119.0 (2)
F1—Cu1—N4	95.15 (6)	N1—C5—C4	122.62 (18)
N1^i^—Cu1—N2^i^	80.32 (7)	N1—C5—C6	114.45 (17)
N1^i^—Cu1—N3	171.30 (7)	C4—C5—C6	122.94 (19)
N1^i^—Cu1—N4	107.82 (7)	N2—C6—C5	114.80 (18)
N2^i^—Cu1—N3	91.34 (7)	N2—C6—C7	110.23 (17)
N2^i^—Cu1—N4	170.90 (7)	C5—C6—C7	134.97 (18)
N3—Cu1—N4	80.32 (7)	C6—C7—C8	103.05 (17)
F1—P1—F2	90.45 (8)	N3—C8—C7	110.27 (17)
F1—P1—F3	88.14 (7)	N3—C8—C9	114.66 (17)
F1—P1—F4	89.73 (9)	C7—C8—C9	135.07 (19)
F1—P1—F5	89.48 (8)	N4—C9—C8	114.25 (17)
F1—P1—F6	179.00 (8)	N4—C9—C10	122.46 (18)
F2—P1—F3	178.59 (8)	C8—C9—C10	123.29 (18)
F2—P1—F4	90.10 (8)	C9—C10—C11	119.1 (2)
F2—P1—F5	90.07 (8)	C10—C11—C12	119.2 (3)
F2—P1—F6	90.55 (8)	C11—C12—C13	118.8 (3)
F3—P1—F4	89.93 (8)	N4—C13—C12	123.6 (2)
F3—P1—F5	89.88 (8)	N1—C1—H1	118.447
F3—P1—F6	90.86 (8)	C2—C1—H1	118.447
F4—P1—F5	179.19 (9)	C1—C2—H2	120.455
F4—P1—F6	90.15 (9)	C3—C2—H2	120.458
F5—P1—F6	90.64 (9)	C2—C3—H3	120.463
Cu1—F1—P1	141.77 (8)	C4—C3—H3	120.456
Cu1^i^—N1—C1	129.86 (15)	C3—C4—H4	120.518
Cu1^i^—N1—C5	112.79 (13)	C5—C4—H4	120.513
C1—N1—C5	117.14 (17)	C6—C7—H5	128.470
Cu1^i^—N2—N3	134.19 (12)	C8—C7—H5	128.475
Cu1^i^—N2—C6	117.43 (13)	C9—C10—H6	120.440
N3—N2—C6	108.34 (16)	C11—C10—H6	120.440
Cu1—N3—N2	134.43 (13)	C10—C11—H7	120.375
Cu1—N3—C8	117.46 (13)	C12—C11—H7	120.382
N2—N3—C8	108.11 (15)	C11—C12—H8	120.612
Cu1—N4—C9	113.20 (13)	C13—C12—H8	120.598
Cu1—N4—C13	129.88 (14)	N4—C13—H9	118.220
C9—N4—C13	116.83 (17)	C12—C13—H9	118.227
N1—C1—C2	123.1 (3)		
			
F1—Cu1—N1^i^—C1^i^	−88.93 (13)	Cu1^i^—N2—N3—C8	177.44 (11)
F1—Cu1—N1^i^—C5^i^	85.58 (10)	Cu1^i^—N2—C6—C5	1.66 (19)
N1^i^—Cu1—F1—P1	65.41 (14)	Cu1^i^—N2—C6—C7	−177.98 (9)
F1—Cu1—N2^i^—N3^i^	93.39 (14)	N3—N2—C6—C5	179.56 (13)
F1—Cu1—N2^i^—C6^i^	−83.83 (10)	N3—N2—C6—C7	−0.08 (19)
N2^i^—Cu1—F1—P1	145.75 (14)	C6—N2—N3—Cu1	179.64 (14)
F1—Cu1—N3—N2	−87.27 (14)	C6—N2—N3—C8	0.04 (18)
F1—Cu1—N3—C8	92.31 (11)	Cu1—N3—C8—C7	−179.67 (9)
N3—Cu1—F1—P1	−122.97 (14)	Cu1—N3—C8—C9	0.68 (19)
F1—Cu1—N4—C9	−91.72 (10)	N2—N3—C8—C7	0.02 (19)
F1—Cu1—N4—C13	84.74 (13)	N2—N3—C8—C9	−179.64 (13)
N4—Cu1—F1—P1	−42.23 (14)	Cu1—N4—C9—C8	−3.48 (18)
N1^i^—Cu1—N2^i^—N3^i^	−179.62 (15)	Cu1—N4—C9—C10	176.67 (11)
N1^i^—Cu1—N2^i^—C6^i^	3.16 (10)	Cu1—N4—C13—C12	−176.39 (12)
N2^i^—Cu1—N1^i^—C1^i^	−178.70 (14)	C9—N4—C13—C12	−0.0 (3)
N2^i^—Cu1—N1^i^—C5^i^	−4.20 (10)	C13—N4—C9—C8	179.56 (15)
N1^i^—Cu1—N4—C9	179.88 (9)	C13—N4—C9—C10	−0.3 (3)
N1^i^—Cu1—N4—C13	−3.67 (14)	N1—C1—C2—C3	0.4 (4)
N4—Cu1—N1^i^—C1^i^	5.49 (14)	C1—C2—C3—C4	−0.4 (4)
N4—Cu1—N1^i^—C5^i^	180.00 (9)	C2—C3—C4—C5	0.3 (3)
N2^i^—Cu1—N3—N2	2.12 (15)	C3—C4—C5—N1	−0.1 (3)
N2^i^—Cu1—N3—C8	−178.30 (11)	C3—C4—C5—C6	179.75 (17)
N3—Cu1—N2^i^—N3^i^	−2.11 (14)	N1—C5—C6—N2	2.1 (3)
N3—Cu1—N2^i^—C6^i^	−179.33 (11)	N1—C5—C6—C7	−178.41 (17)
N3—Cu1—N4—C9	3.01 (10)	C4—C5—C6—N2	−177.79 (17)
N3—Cu1—N4—C13	179.47 (14)	C4—C5—C6—C7	1.7 (4)
N4—Cu1—N3—N2	178.43 (15)	N2—C6—C7—C8	0.1 (2)
N4—Cu1—N3—C8	−1.99 (10)	C5—C6—C7—C8	−179.44 (19)
F2—P1—F1—Cu1	−17.33 (14)	C6—C7—C8—N3	−0.1 (2)
F3—P1—F1—Cu1	162.63 (13)	C6—C7—C8—C9	179.50 (18)
F4—P1—F1—Cu1	−107.43 (14)	N3—C8—C9—N4	2.0 (3)
F5—P1—F1—Cu1	72.73 (14)	N3—C8—C9—C10	−178.19 (14)
Cu1^i^—N1—C1—C2	−174.59 (12)	C7—C8—C9—N4	−177.58 (19)
Cu1^i^—N1—C5—C4	175.38 (11)	C7—C8—C9—C10	2.3 (4)
Cu1^i^—N1—C5—C6	−4.49 (18)	N4—C9—C10—C11	0.2 (3)
C1—N1—C5—C4	0.1 (3)	C8—C9—C10—C11	−179.59 (15)
C1—N1—C5—C6	−179.75 (15)	C9—C10—C11—C12	0.1 (3)
C5—N1—C1—C2	−0.3 (3)	C10—C11—C12—C13	−0.4 (3)
Cu1^i^—N2—N3—Cu1	−3.0 (3)	C11—C12—C13—N4	0.4 (4)

Symmetry code: (i) −*x*, −*y*+2, −*z*+1.

### Hydrogen-bond geometry (Å, º)

**Table:**

*D*—H···*A*	*D*—H	H···*A*	*D*···*A*	*D*—H···*A*
C3—H3···F3^ii^	0.95	2.31	3.257 (3)	175
C11—H7···F2^iii^	0.95	2.54	3.451 (3)	162
C12—H8···F5^iv^	0.95	2.60	3.456 (3)	150
C13—H9···F3^iv^	0.95	2.52	3.226 (3)	131

Symmetry codes: (ii) −*x*−1, *y*−1/2, −*z*+1/2; (iii) −*x*+1, −*y*+2, −*z*; (iv) *x*+1, *y*, *z*.
